# Ethics

**Published:** 1869-08

**Authors:** J. F. Johnson


DENTAL REGISTER.

VOL. XXIII.] AUGUST, 1869. [NO. 8.

### Original Communications.

### ETHICS.

BY J. F. JOHNSON, D. D. S.

[Read before the Indiana Dental Association.]

*Mr. President and Gentlemen of the Indiana State Dental Association :*

I desire to call your attention for a short time to the consideration of the following question: Is the Code of Ethics which has been embodied into and made a part of the Constitution of the Indiana State Dental Association to be ignored, or shall we not hereafter demand a closer observance of its requirements, and thereby secure a better professional standing of membership in our State Association?

What I am about to say may be regarded by some as illiberal and arrogant, but waiving all fears of that sort and trusting to the candid and matured judgment of the experienced gentlemen present, I venture to assert the opinion that the generally lax system that prevails in the examination of candidates for membership, the careless observance of the requirements of the Code of Ethics, and the general anxiety apparently to increase the strength of our professional edifice through members, rather than in the quality of the materials, has a tendency to retard, rather than advance the true interests of Dentistry, as well as to destroy in part the usefulness of all such bodies, by degrading them in the opinion of the profession and the world. There is, perhaps, no more true aphorism than this, that the true index to the character of men is found in their associations. We can hardly, therefore, expect the hearty co-operation of the best materials in the profession, so long as we continue to make but little, if any distinction. It is quite as probable that the high-toned professional man would as naturally shrink from indiscriminate associations professionally, as do the same class in literary or polite circles of society.

There can be no doubt that the admission of a candidate to membership in this or any other Dental Association, is a virtual indorsement of him as a genuine representative, a qualified member of the profession. In brief, it is a recognition of him, full and complete, so far as the practice of Dentistry is concerned, and it should be so too; and that indorsement should be of relative value to each member as is the diploma to the graduate of a college, and such would be the case if the proper care and nerve was used in examining applicants and reporting upon applications by the Board of Censors, and members would have firmness enough to vote their true sentiments, without affection or favor, malice or jealousy; in other words, if we would but *live up* to the Constitution and By-laws we have adopted for our government. We have, perhaps, all been equally in fault in this particular. I do not know of a single instance in this Association where the applicant has been rejected, or where the examination amounted to any thing more than a mere farce. If we have nothing but the best among us, we owe it, by far, more to good luck than carefulness on our part. I must not be understood as condemning any one even by implication. I am but speaking of general principles. If our associations cut loose from the rubbish and admit only well qualified, at any rate legitimate practitioners, we should then be acting in harmony with the generally expressed desire of nearly every well qualified man in the profession, and the vigorous stimulus this course would give to Dental education, would soon cause Dental Colleges to be better patronized, Dental literature more carefully read, and the term of pupilage adhered to with greater uniformity, with more general satisfaction and with more lasting benefit to all concerned. But it has been said that Dental associations are educators, which is true, to a certain extent, for we are all learners, and the popular indea has been to take cordially by the hand all who present themselves, and try to draw them up after us to the "hights of Dental science." This is a sort of poetical piece of justice and benevolence and very well in its place but the place is not a Dental Association. Very likely that sort of thing was necessary some time past and was then perhaps true philanthropy, perhaps good policy. It would not be so in my judgment now. The necessity no longer exists. The Dental Associations now are called upon to give tone, and by unity of purpose support and reorganize the worthy practitioner as far as may be, and not to bolster up, as is now unfortunately by far too often the case, the merest apology for a Dentist. Therefore, I think if we expect to increase the usefulness of our associations, we must begin by making them entitled to the respect and active support of the best among us; in brief, "not to put too fine a point upon it," I am in favor of a select Dental Association, conducted upon the same principles substantially that controls good society every where, or good medical or legal associations any where. Dental Associations, therefore, although educators, ought not to be relied upon for the purpose of fitting students or others for practice, or in any way to supersede the colleges in this legitimate work. They should be auxiliary thereto and create, by their high position, a still greater demand for a collegiate course from all those who desire to enter their portals. If, then, the leading portion of the profession could be thoroughly united and have fixed views and rules as to length of pupilage, course of studies, etc., they would soon draw after them the major part of those who will long continue to practice, for, in this day of enlightenment and of opportunities to be informed, no countenance should or would long be given to quacks or incompetents.

The Convention of the College Faculties in Philadelphia was a very important advance step in Dental education. It can not but be gratifying to every regular graduate of any of them to learn that the almost reckless graduating for a monied consideration, by honorary degrees, has been by nearly all of them abandoned, and it is not difficult to see, at least I venture to predict, that any institution of Dental education that will continue to do as heretofore, or withholds its influence from this wholesome movement will not long receive the support of the educated and influential members of the profession, nor will much value attach to its diplomas. However, but little good, comparatively speaking, can be expected from the high position taken by our Dental Colleges, whilst our associations continue to take into full fellowship every self-styled D. D. S. that presents himself. The fact is, as a profession, we are beset by a brazen set of fellows, who are sometimes taken into respectable offices and who take mortal offense if not introduced to all visitors as doctor. Members here agree not to take a student for a shorter term than two years, which is in reality not long enough; but how many live up to it? Why, we know of their being frequently started out as full blown roses in the profession in from six to twelve months, and the parties so doing have the hardihood to sometimes complain of cheap Dental competition, and tell you very gravely and lackadaisically that the profession is going to the dogs, which is correct, so far as they are concerned. Such men should be made to feel that they have not acted in good faith with us, but have violated the Code and, instead of being elevated to positions of trust and honor, as is sometimes done, should receive the condemnation of the Association. This style of man, this hot bed growth and grower, with more brass than manners, often calls and claims the attention and hospitalities of gentlemen. He rushes into your office and private surgery, not waiting for the formality of being asked; strides up to your chair, if you are engaged, to observe you whilst operating, regardless of the feelings of yourself or patient. He perhaps may attempt an apology by saying he will not take your time from business, but talk to you then and there, and very likely tell you he is taking items. This sort of individual has generally several assistants at home, who are left perfectly overwhelmed with work and are at that time making frantic efforts, night and day, to get through engagements. He had just to tare himself away, or slip off and take a little time to recuperate his wasted energies. We talk longingly and admiringly of dignity, professional etiquette and all that, but we must select for our students young men capable of such sentiments, and for our associates such as have these traits, if we expect to realize our desires.

We have recently been besieging our Legislature with a view to get a bill passed to regulate the practice of Dentistry. Although not successful in bringing it to a final vote on account of a press of other matter that had precedence, we were yet received with uniform courtesy and kindness, and so far as we could learn, with but very few exceptions, the restrictive law proposed was looked upon with favor. But with what propriety can we importune our legislators to take an active interest in this matter, if we who are far more concerned and whose especial business it is, fail to do for ourselves what is within our power and can reasonably be expected of us? We ask the Legislature to protect us by law from the machinations of quacks, and do absolutely nothing to guard ourselves by the means that is at our command. This is unreasonable, to say the least; therefore, I take this position, if we wish to see a law passed regulating Dental practice, to be consistent we must take a decided stand in a fixed antagonism in our societies to all quacks and merely mercenary practitioners, and then if all worthy Dentists would unite with us, which they would be much more inclined to do under such an organization, the mere charlatan would be powerless to drag us down to his level in any case. We must have respect for ourselves if we wish others to respect us. Our societies must be careful then as to whom they admit to full membership, not losing sight of the fact that this membership is a full indorsement before the world. The old self made Dentist, who has plodded his way wearily through tribulation, without any of the advantages now within the reach of all will be found ready and anxious to subscribe to these rules, and none will recognize their entire justness quicker than he. It is the Dentist of to-day who has no occasion and no excuse to go among the public unprepared, who should hesitate long before taking that step; and it is the merely unprincipled charlatans, anxious to make money easily, as they suppose, and caring but little how dishonestly, who will be found quarreling over this classification. Hereafter then, the graduate only of a regularly organized and chartered College, or the practitioner of from six to ten years standing, should be eligible or entitled to full fellowship in the profession. We owe this to the Colleges, to society at large and ourselves. The utmost courtesy, however, should be exhibited to all worthy young men who are making laudable and honorable efforts to complete their course. The want of sufficient means deters many from following the bent of their inclinations. We should encourage such by employing them when we can, and urge them to abstain from general practice independently until they have been found fit, by a regular faculty, to discharge its varied, mportant and responsible duties creditably and honorably; and if such, or any others who are not eligible to membership are present and seeking to be benefited by our discussions and deliberations, let us invite them heartily, cordially and kindly to sit with us, but let us cease to make active members of any such persons until their course is completed. Then if we take this position we can, with far more courage and a better prospect of success, appeal to the Legislature and the communities there represented, until the justice and necessity of a restrictive law will ultimately be demanded by others than ourselves and receive no serious opposition. But if even the Legislature should unfortunately fail us in this respect, we will have established the line of demarkation ourselves so thoroughly that the people will soon learn to observe and profit by it. Thus we shall gradually and surely educate the public up to a point wherein they can use some discrimination and create a status that the intelligent portion will not be slow to observe. Of course this can not be done entirely, but sufficiently to make the real Dentist feel much more comfortable in his position; he will feel that he is better appreciated and recognized as a member of a dignified and respectable profession. That the public do need more information on this point we all agree and many have had cause to regret. I have now in my possession a letter from a worthy gentleman, who finds his business almost ruined by the devices of what he styles "these cheap Dentists," who, he says, make it a point to extract the natural teeth, good or bad, when they can, and replace them with artificial, greatly cheaper than a good operator can fill and save them, and this community have not cultivation enough as yet to know any better; at least they are not enlightened on this subject. Now, if such Dentists as just spoken of applied for admission into this Association, in my judgment, they ought to be rejected. Or, if we have any such now as members they ought to be expelled. The fact is, young men are too much inclined to consider themselves Dentists as soon as they can fit up and vulcanize a set of teeth, and that, too often in a very inferior manner. Their preceptors are frequently to blame for this; instead of placing the literature of the profession in their hands, their students are more frequently supplied with an impression, some old artificial teeth, &c. and they are told to go to work and "put up a job." Gentlemen, this is all wrong; we shall retrograde, rather than advance, under such a system. We do ourselves injustice and the students also, and the communities in which these individuals may eventually locate, a still greater injustice. At first blush it may appear to young students that I am in antagonism with them—that I am an enemy to such. I am sure that on reflection they will arrive at a different conclusion. I am well convinced, however, that any respectable young man would not care to enter a profession that guarded its doors less carefully than the course indicated in these remarks would have them.

I am aware, nevertheless, that I have been traveling over very dangerous ground in my efforts to find a direct road to a more elevated position for Dentistry as a profession. I have done this fearlessly, however, having no ill-will toward any individual member of the profession or association, neither have I had reference to any one in the Association, for I trust all would be found worthy after the most rigid examination. These thoughts are expressed candidly, without enmity, affection or favor to any one, and for the common good. The motive being pure, I shall not fear that any ill-will toward myself will grow out of it, and if it does after this frank avowal and disclaimer of any evil intent, I shall not care.

